# Improving lung point-of-care ultrasound (POCUS) training and accreditation - a multidisciplinary, multi-centre and multi-pronged approach to development and delivery using the action learning process

**DOI:** 10.1186/s12909-024-05653-2

**Published:** 2024-07-02

**Authors:** Mark ZY Tan, Annemarie Brunswicker, Harry Bamber, Alistair Cranfield, Evangelos Boultoukas, Sam Latif

**Affiliations:** 1 ST7 anaesthesia and intensive care medicine trainee, Health Education England Northwest, Manchester, UK; 2 NIHR academic clinical fellow, Humanitarian and Conflict Response Institute, University of Manchester, Manchester, UK; 3 ST8 thoracic surgery trainee, Health Education England Northwest, Manchester, UK; 4 ST3 anaesthesia trainee, Health Education England Northwest, Manchester, UK; 5 Consultant in anaesthesia and intensive care medicine, Salford Royal NHS Foundation Trust, Salford, UK; 6 Consultant in intensive care medicine, North Manchester General Hospital, Manchester, UK; 7 Consultant in anaesthesia and intensive care medicine, Stockport NHS Foundation Trust, Stockport, UK

**Keywords:** POCUS, Chest drain, Action learning, Accreditation, Education, Lung ultrasound, Credentialing, Intensive care, Emergency medicine, Acute medicine

## Abstract

**Background:**

Point-of-Care Ultrasound (POCUS) consists of a range of increasingly important imaging modalities across a variety of specialties. Despite a variety of accreditation pathways available in the UK, lung POCUS training remains difficult to deliver and accreditation rates remain suboptimal. We describe a multidisciplinary, multi-centre, and multi-pronged approach to lung POCUS education within a region.

**Methods:**

A survey was conducted in a region. From these results, bottlenecks were identified for improvement. We utilised key stages in an established accreditation pathway, and the Action Learning process. Analysing participant feedback, consensus amongst the team, regional educational needs, and leveraging the expertise within the faculty, we implemented several solutions which were multidisciplinary, multi-centre, and multi-pronged. We also set up a database across several accreditation pathways to facilitate supervision and assessment of rotational trainees.

**Results:**

Utilising the Action Learning process, we implemented several improvements at elements of the lung ultrasound accreditation pathways. An initial regional survey identified key barriers to accreditation: lack of courses (52%), lack of mentors (93%), and difficulty arranging directly supervised scans (73%). A multidisciplinary team of trainers was assembled. Regular courses were organised and altered based on feedback and anecdotal educational needs within the region. Courses were set up to also facilitate continuing professional development and exchange of knowledge and ideas amongst trainers. The barrier of supervision was removed through the organisation of regular supervision sessions, facilitating up to fifty scans per half day per trainer. We collected feedback from courses and optimised them. Remote mentoring platforms were utilised to encourage asynchronous supervision. A database of trainers was collated to facilitate triggered assessments. These approaches promoted a conducive environment and a commitment to learning. Repeat survey results support this.

**Conclusion:**

Lung ultrasound accreditation remains a complex educational training pathway. Utilising an education framework, recruiting a multidisciplinary team, ensuring a multi-pronged approach, and fostering a commitment to learning can improve accreditation success.

**Supplementary Information:**

The online version contains supplementary material available at 10.1186/s12909-024-05653-2.

## Introduction

Point-of-Care Ultrasound (POCUS) consists of a range of increasingly important imaging modalities across a variety of specialties. The COVID19 pandemic highlighted the importance of the lung ultrasound [1, 2]. Yet, lung POCUS training remains difficult to deliver. This report describes a multidisciplinary, multi-centre, and multi-pronged approach to developing and delivering lung POCUS training in a region.

One of the most established POCUS training pathways in the UK is FUSIC heart (previously known as Focused Intensive Care Echocardiography (FICE)). Unfortunately, accreditation rates across this (and other modalities like lung POCUS) are suboptimal [3]. Currently, lung POCUS features in several accreditation pathways in the UK: Focused Ultrasound in Intensive Care (FUSIC) from the Intensive Care Society [4], Focused Acute Medicine Ultrasound (FAMUS) from the Society of Acute Medicine [5], and the Royal College of Emergency Medicine POCUS curriculum (RCEM POCUS) [6]. In addition, the British Thoracic Society have published a clinical statement on the use of ultrasound for pleural procedures, and their stance on training for lung ultrasound [7]. Lung POCUS is a standalone module for FUSIC and FAMUS, but it is part of the shock module in RCEM POCUS, which also includes focused echocardiography. Alongside lung POCUS, BTS and FUSIC pathways include demonstration of US-guided chest aspiration and/or drainage, while the FAMUS pathway includes marking for drainage, but doesn’t specifically require candidates to perform aspiration or drainage. RCEM POCUS curriculum does not specify chest drainage either, but this is included in wider training requirements. Despite their differences, most accreditation pathways contain key elements: (1) initial learning via a course (e-learning or face-to-face), (2) directly supervised scans, (3) indirectly supervised scans, and (4) a triggered assessment (Table 1). The practical assessment is initiated by the candidate after completion of logbook (thus triggered), consists of performing and reporting a scan on a patient (real or simulated), and is ideally assessed by someone who is not the main trainer for the candidate.

**Table 1:** UK lung ultrasound accreditation pathways

**Elements**						**Our approach**
**Course**	Course	Course	Course	Course	Course	Course with pre-course material
**Directly supervised scans**	10	5	10	eFAST: 25 Shock: 25	50	Regular half-day supervision sessions on the intensive care unit in three hospitals
**Indirectly supervised scans**	20	20	30			Remote mentoring platform e.g. SonoClipShare
**Assessment**	Triggered	Triggered or summative directly observed procedure	Triggered	Work-based assessment or triggered	Evidence review	Assessment facilitated via regional trainer database. Trainer network across several hospitals.
**Chest drains**	Optional	Mandatory	Not stated	Included in wider curriculum	Not stated	Directly Observed Procedure signed during course.
**Continuing development**	Regular practice	Further 50 scans to become trainer	3-year renewal	Regular practice		Paired trainers during courses.

*FUSIC* Focused ultrasound in intensive care, *BTS* British Thoracic Society, *FAMUS* Focused acute medicine ultrasound, *RCEM* Royal College of Emergency Medicine, *CACTUS* Children acute ultrasound. Our approach = summary of interventions discussed in paper

The Northwest of England is a large training deanery responsible for the postgraduate education and training of 8000 doctors [8]. It hosts 12 specialty schools, including intensive care medicine, anaesthetics, medicine, emergency medicine, and others. Before the launch of FUSIC lung, and before the start of this project, the Northwest had good accreditation rates for FICE. Unfortunately, the rates for the other POCUS modalities, including lung ultrasound, were low. Amongst physiotherapists, however, there has been rapid increase in successful accreditation for FUSIC lung [9], which is also reflected in medical doctors discussed in this paper.

## Methods

A baseline qualitative survey, via Google forms, was conducted in the region between September to November 2018 (Table 2). This was self-administered online, and participation was voluntary. It was disseminated through regional training channels (focusing on intensive care medicine) including email and text messaging groups. The survey was designed to target specific elements of the lung POCUS accreditation pathway (specifically FUSIC). This sought to identify key points within the accreditation pathway candidates struggle to complete.

**Table 2:** Summary of survey results (2018)

**Respondent characteristics**		2018		2024	
**Role**		*n*	%	*n*	%
**Anaesthetics/ICM trainee**		14	50%	4	20%
**ICM trainee**		5	17.9%	5	25%
**Anaesthetics trainee**		3	10.7%	2	10%
**EM/ICM trainee**		2	7.1%	1	5%
**ICM consultant**		2	7.1%	1	5%
**Acute Medicine/ICM trainee**		1	3.6%	3	15%
**Anaesthetics consultant**		1	3.6%		
**EM trainee**				3	15%
**Acute Medicine trainee**				1	5%
**Total**		28	100%	20	100%
**Hospital**					
**Manchester Royal Infirmary**		5	17.9%	3	15%
** Pennine Acute Hospital**		4	14.3%	1	5%
** Wythenshawe Hospital**		3	10.7%	4	20%
** Salford Royal Hospital**		3	10.7%	4	20%
** Lancashire Teaching Hospitals**		3	10.7%	2	10%
** Royal Bolton Hospital**		2	7.1%	2	10%
** Royal Manchester Children’s Hospital**		2	7.1%		
** Stockport NHS Foundation Trust**		1	3.6%		
** University Hospitals Morecambe Bay**		1	3.6%		
** Wirral University Hospital**		1	3.6%		
** Alder Hey Hospital**		1	3.6%		
** Royal Liverpool Hospital**		1	3.6%		
** Arrowe Park Hospital**		1	3.6%		
** Blackpool, Fylde, Wyre Hospitals**				1	5%
** North Manchester General Hospital**				1	5%
** East Lancashire Teaching Hospital**				2	10%
** Total**		28	100%	20	100%
		2018		2024	
		*n*	%	*n*	%
**Access to lung US mentors**	**Yes**	2	7.0%		
	**No**	26	93.0%		
**Ease of booking onto FUSIC course**	**Very easy**	1	4.3%	1	5.0%
	**Easy**	2	8.7%	6	30.0%
	**Neither easy nor difficult**	8	34.8%	4	20.0%
	**Difficult**	8	34.8%	5	25.0%
	**Very difficult**	4	17.4%	1	5.0%
**Achieving directly supervised scans**	**Very easy**	0	0.0%	0	0.0%
	**Easy**	1	4.3%	2	10.0%
	**Neither easy nor difficult**	5	21.7%	5	25.0%
	**Difficult**	9	39.1%	6	30.0%
	**Very difficult**	8	34.8%	5	25.0%
**Reviewing for indirectly supervised scans**	**Very easy**	0	0.0%	2	10.0%
	**Easy**	0	0.0%	2	10.0%
	**Neither easy nor difficult**	3	13.6%	4	20.0%
	**Difficult**	10	45.5%	6	30.0%
	**Very difficult**	9	40.9%	4	20.0%
**Triggered assessment**	**Very easy**	0	0.0%	1	5.0%
	**Easy**	0	0.0%	2	10.0%
	**Neither easy nor difficult**	4	18.2%	2	10.0%
	**Difficult**	9	40.9%	5	25.0%
	**Very difficult**	9	40.9%	2	10.0%
				Barriers improved	
**Top 3 barriers to accreditation**	**Availability of courses**	14		11	55%
	**No mentors in base hospital**	14		5	25%
	**Directly supervised scans**	11		6	30%
**Top 3 options for improvement**	**Regular POCUS courses**	24			
	**Improve availability of trainers across hospitals**	19			
	**Remote supervision**	18			

We utilised the Action Learning process for this project [10]. This is a problem-solving approach characterised by cycles of action and reflection. The key stages in the process are (1) An important and complex problem, (2) A diverse problem-solving team, (3) An environment that promotes curiosity, inquiry, and reflection, (4) Talk that becomes action and solutions, and finally, (5) A collective commitment to learning. Action Learning has been used in a variety of educational contexts, including medical education. It has been shown to be particularly effective for supporting collaborative and cooperative approaches between disciplines and sectors [11, 12].

Since the different accreditation pathways share many key elements (see introduction), we focused on the FUSIC pathway. We mapped out the key stages of accreditation, and designed interventions to maximise opportunities for candidates to achieve progression at each stage. We collected and analysed feedback from the courses, integrating them with anecdotal observations from senior clinicians and trainers in the region, and together with the emergence and desire for POCUS training from other specialties and towards different accreditation pathways, we continued to build a multidisciplinary approach to lung POCUS accreditation.

Whilst this project was not classified as research, we adhered to the UK Research and Innovation principles of ethics framework [13]. The risks of performing ultrasound scans are negligible, but the potential benefits are significant [14, 15]. Trainers are all highly experienced clinicians regulated by the General Medical Council’s duties to uphold patients’ rights and dignity. Verbal consent is obtained from patients whenever possible. Scanning stopped if discomfort or deterioration was recognised. None of the trainers have conflict of interests, and all training scans are reviewed by our team of trainers, who also maintain communication with clinical teams should an unexpected finding be noticed.

## Results

The results below are presented in the format of the Action Learning process and framed within the FUSIC accreditation pathway. A further summary of these actions is presented in Table 3.

**Table 3:** Action learning process during each lung ultrasound element

**Action learning process/POCUS pathway**	**Course**		**Directly supervised scans**		**Indirectly supervised scans**		**Triggered assessments**	
**An important and complex problem**	2018	No courses	2018	Lack of trainers	2018	Difficulty in arranging supervision. In-person meeting cumbersome.	2018	Lack of trainers
	2019	Full FUSIC course (lung, abdomen, DVT)						
**A diverse problem solving team**	2022	MDT lung US and chest drain course (ICM, EM, Resp, CTSurg)						
**An environment that promotes curiosity, inquiry, and reflection.**	2022	Paired trainers from different disciplines						
**A requirement that talk be converted into action and, ultimately, a solution.**	2022	Course feedback: pre-course materials made available.	2019	AMU supervision sessions. Difficult logistics. Too many candidates per trainer	2022	Remote mentoring platform.		
			2023	Half-day ICU supervision sessions. 5 candidates per trainer. 10 scans each = 50 scans per session.				
**A collective commitment to learning.**					2024	Shared remote mentoring platform account for exchange of knowledge amongst trainers.	2022	Multidisciplinary trainer database
							2024	Systematic data collection for regional trainer database

*Abbreviations*: *AMU* Acute medicine unit, *CTSurg* Cardiothoracic surgery, *EM* Emergency medicine, *FUSIC* Focused ultrasound in intensive care, *ICM* Intensive care medicine. *MDT* Multidisciplinary team

The first vertical column depicts stages in the Action Learning process. Horizontal rows follow the general elements in lung ultrasound accreditation pathways. Table elements highlight cycles of improvement within each stage of the Action Learning process

### An important and complex problem

**Fig. 1:**
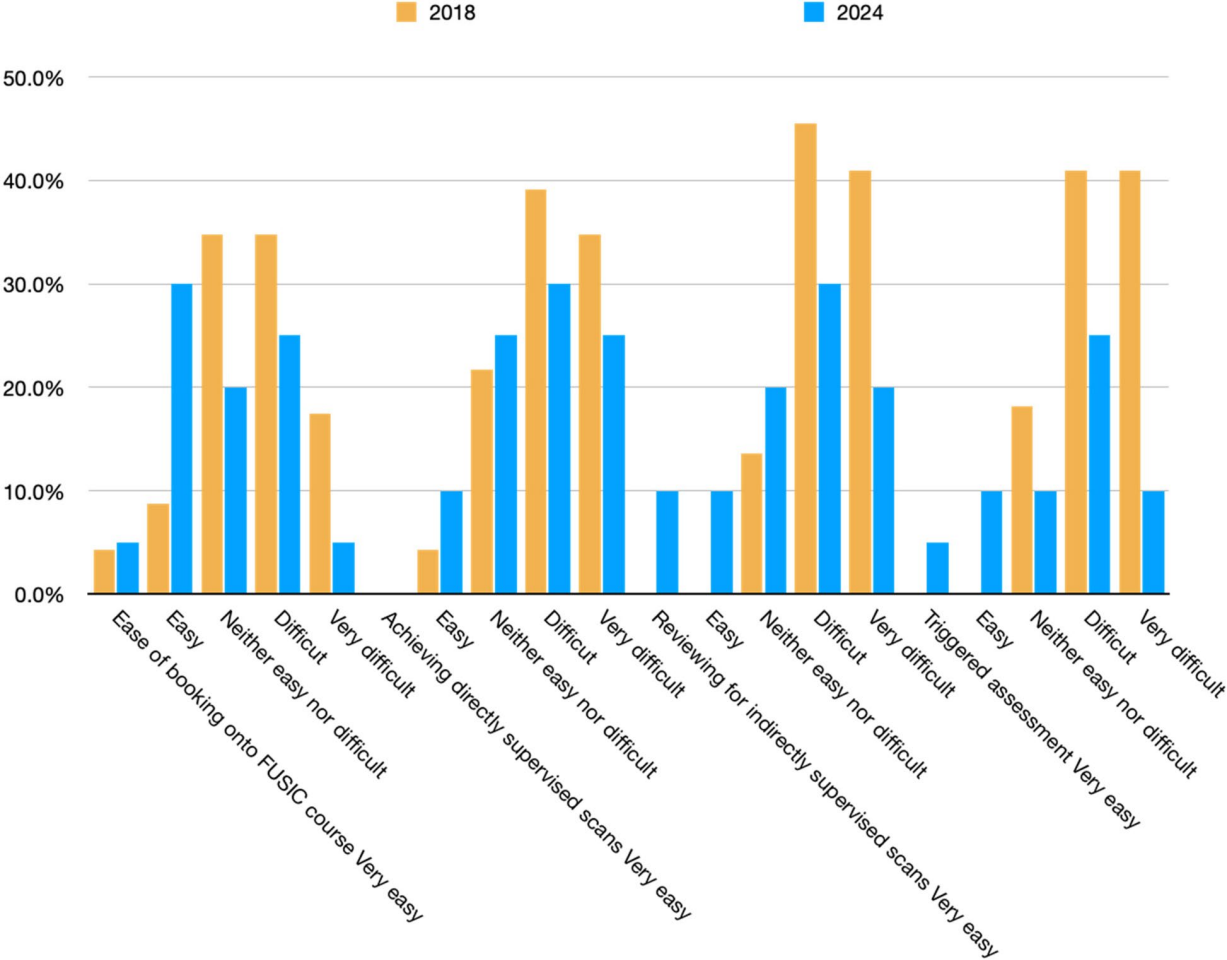
Survey results from 2018 and 2024.

There were 28 respondents for the survey between September to November 2018. Table 2 and Fig. 1 summarise their characteristics and key findings. Although 19 (68%) respondents had access to FUSIC heart mentors in their hospitals, 26 (93%) did not have access to trainers in other modalities (lung, abdomen, deep vein thrombosis). 12 (52%) found it difficult/very difficult to book onto a FUSIC lung course, and 17 (74%) found it difficult/very difficult to obtain their first ten supervised scans. The top three barriers to FUSIC lung accreditation were (1) lack of availability of courses, (2) lack of mentors, and (3) difficulty in arranging directly supervised scans. The lack of mentors and education is a common theme amongst other professional groups too [9]. Through this survey, we not only identified an important and complex problem, but also highlighted several areas which may have practical solutions. The FUSIC pathway was used as a template, acknowledging that most other pathways share many similar elements but differ in the overarching governance processes and recommended numbers.

**Fig. 2:**
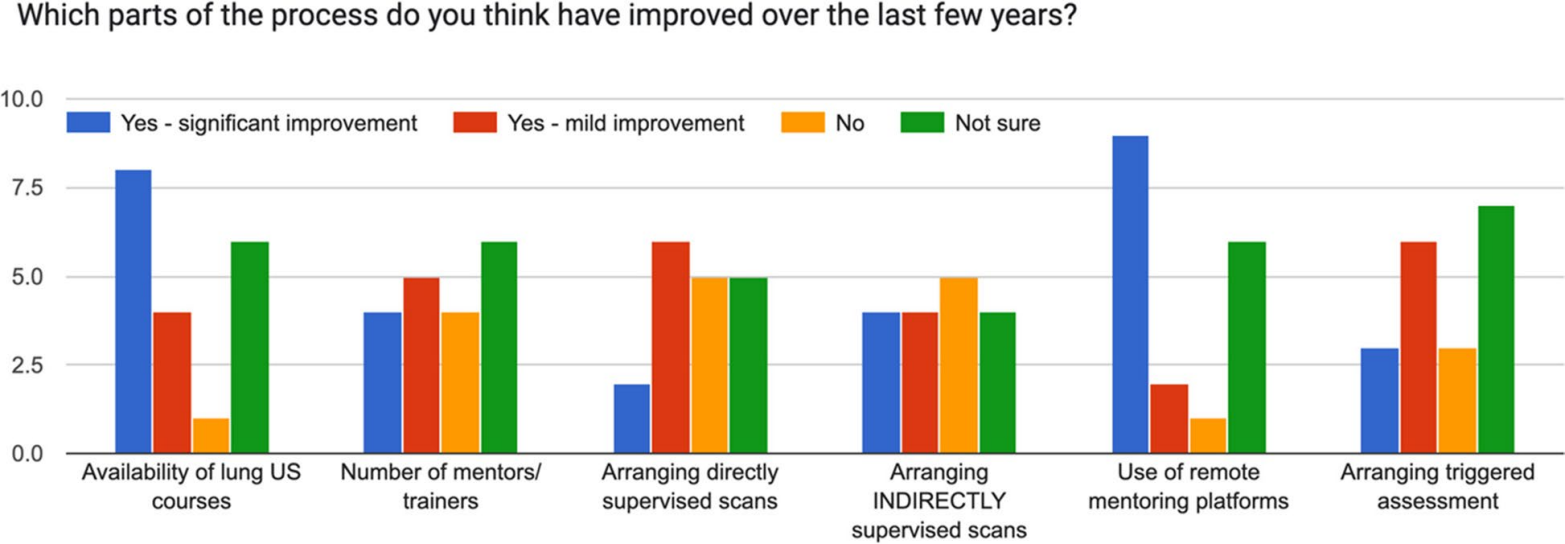
Survey data showing improvements in the elements of the pathways

A survey was repeated in 2024 (Table 2, Fig. 1). There were 20 respondents. 11-12 (55-60%) thought there was better availability of lung US courses and 6 (30%) found it easier to arrange directly supervised scans (Figs. 1 and 2). There were fewer respondents who found elements of the pathways difficult or very difficult (Fig. 1). 45% thought there have been mild to significant improvements in the use of remote mentoring platforms (Fig. 2).

### A diverse problem solving team

The first FUSIC course in the Northwest of England was delivered in February 2019. This course covered the modalities of lung, abdomen, deep vein thrombosis, and vascular access. 14 candidates attended this course. Several issues were noted. First, we only captured Intensive Care Medicine trainees (apparent from registration data), despite the increased emphasis on POCUS within the specialities of Emergency Medicine (RCEM POCUS), Acute Medicine (FAMUS) [16], and Respiratory Medicine (BTS pathway). Second, there was anecdotal evidence of desires for procedural skills within the region, which corresponded with the curriculum requirements of chest drain insertion for Emergency Medicine, Respiratory Medicine, and Intensive Care Medicine. This information was obtained from discussions with programme directors, consultants, and trainees in the region. Third, in 2019 and 2023, FUSIC and FAMUS respectively split into their modular components rather than a full suite of POCUS modalities.

**Fig. 3:**
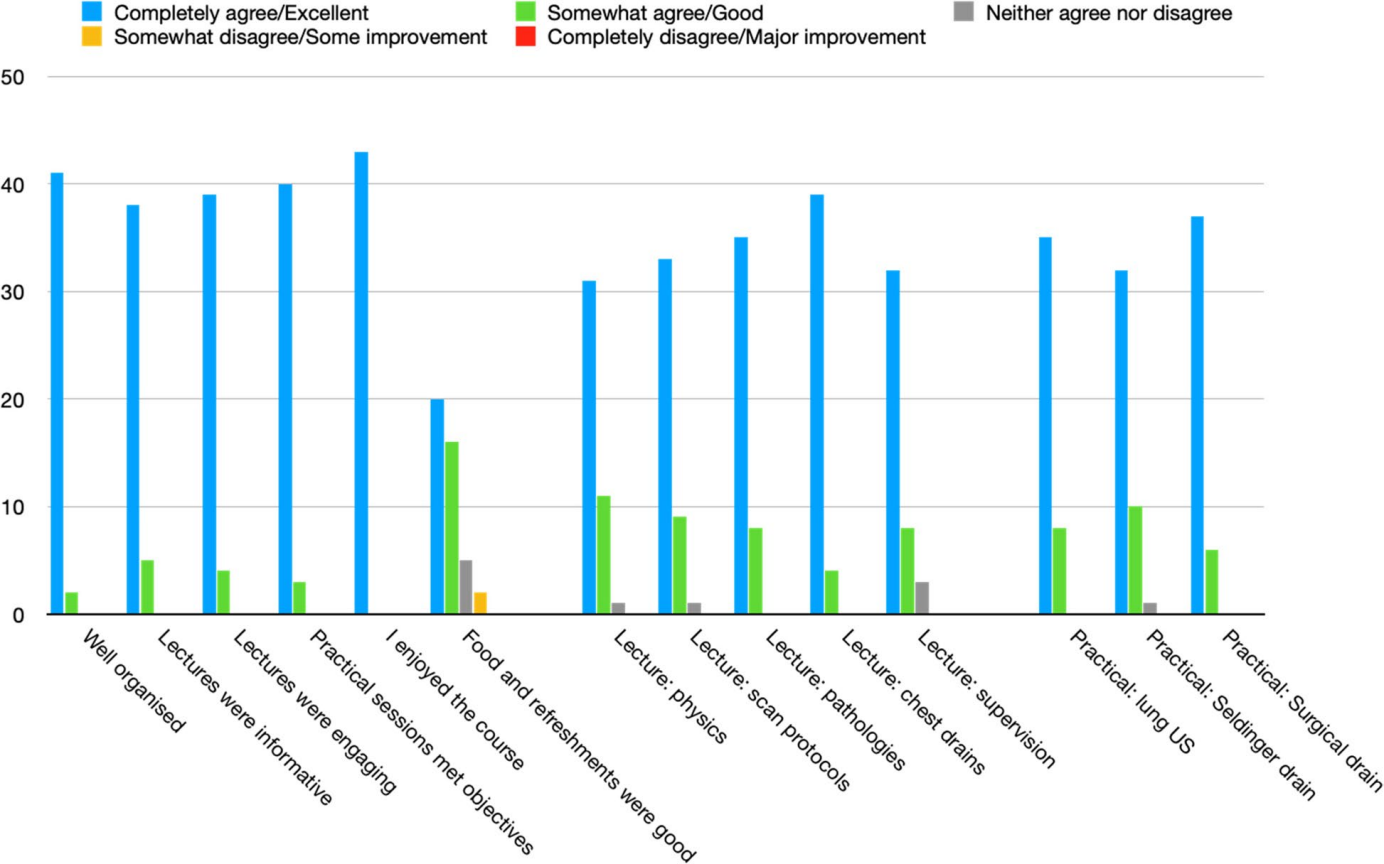
Course feedback

With this knowledge, we redesigned the course into a one-day multidisciplinary lung ultrasound and chest drain course with faculty from intensive care medicine, acute medicine, respiratory medicine, emergency medicine, and cardiothoracic surgery. We actively recruited faculty across FUSIC, FAMUS, RCEM POCUS and BTS training pathways. This course was then delivered in October 2022, following the almost two-year hiatus in training opportunities due to the COVID19 pandemic (they are delivered twice a year now). Thirty candidates attended this course from a variety of specialities from intensive care medicine, emergency medicine, medicine, anaesthetics, surgery, and paediatrics, and from a wide range of seniorities. This was achieved by dissemination through the various specialties, facilitated by the multidisciplinary faculty. The course consisted of several lectures covering essential ultrasound and chest drain knowledge, and three practical sessions (lung ultrasound, seldinger chest drain insertion, surgical chest drain insertion). A healthy volunteer was used for the ultrasound scanning, while a mix of plastic models and pig thoraces were used for the chest drains. Each practical session lasted an hour, with at least two trainers to a group of ten candidates. Feedback from this reformatted course was very positive (Table 4, Fig. 3). This is now run regularly.

**Table 4:** Course feedback (2022-2023)

	**Completely agree/Excellent**	**Somewhat agree/Good**	**Neither agree nor disagree**	**Somewhat disagree/some improvement**	**Completely disagree/major improvement**
**Well organised**	41	2	0	0	0
**Lectures were informative**	38	5	0	0	0
**Lectures were engaging**	39	4	0	0	0
**Practical sessions met objectives**	40	3	0	0	0
**I enjoyed the course**	43	0	0	0	0
**Food and refreshments were good**	20	16	5	2	0
**Lecture: physics**	31	11	1	0	0
**Lecture: scan protocols**	33	9	1	0	0
**Lecture: pathologies**	35	8	0	0	0
**Lecture: chest drains**	39	4	0	0	0
**Lecture: supervision**	32	8	3	0	0
**Practical: lung US**	35	8	0	0	0
**Practical: Seldinger drain**	32	10	1	0	0
**Practical: Surgical drain**	37	6	0	0	0
**Selected free text feedback from**					
**Oct 22**	Ideally, lectures would be sent as pre course learning material to orient candidates with the new information as it seemed abit too much to take in at one setting.				
**Oct 22**	PowerPoint handout re practical tips and US physics principles would be great thank you				
**Oct 22**	Longer on scan protocol & less on physics. More on abnormal findings				
**Oct 22**	Ideally, lectures would be sent as pre course learning material to orient candidates with the new information as it seemed abit too much to take in at one setting.				
**Oct 22**	Excellent course, as a fairly inexperienced trainee the instructors were able to tailor the teaching in group sessions to suit all members of the group with varying levels of experience-this was very much appreciated!				
**Oct 22**	Friendly welcoming faculty, good interaction				
**Mar 23**	Thank you so much for the effort that went into this course. It was an area in which I had low confidence but this course has been an immense help!				
**Mar 23**	The full day was brilliant - very informative and engaging, practical sessions especially were well organised and was the most useful part of the day for me - I learnt a lot and the facilitators were all great.				
**Mar 23**	I thoroughly enjoyed the course and was amazed at how many supportive staff were on hand to teach / help / answer questions. The post course support is fantastic…

### An environment that promotes curiosity, inquiry, and reflection

Along with the diverse faculty at the course, there was a need to create a supportive training environment. Literature has highlighted the benefit of exchanging knowledge and skills between trainers [17], and the importance of maintaining effective relationships between different disciplines [18]. We utilised a small number of lectures for the courses. They transmit important technical information and fulfil the requirements of the various accreditation pathways. The lectures are taught by clinicians from different specialties, ensuring a multidisciplinary environment for learning.

For the practical stations in the courses, we ensured each group was taught by two trainers. Usually, a junior faculty is paired with a more senior trainer, promoting the development and exchange of teaching techniques. A teaching pair from different specialities also facilitates the exchange of skills, knowledge, and perspectives. For example, techniques used by cardiothoracic surgeons for chest drain insertion may differ from the usual practice of emergency physicians. Likewise, ultrasound location prior to chest drain insertion is almost always done by respiratory physicians but seldom by cardiothoracic surgeons. Through the pairing, such skills can be exchanged to promote patient safety and trainers can develop a wide range of teaching techniques.

### A requirement that talk be converted into action and, ultimately, a solution

From the survey, it was clear that mentors were in short supply. As a result, directly supervised scans were tight bottlenecks in the accreditation pathway. To address this, we initially developed an additional day of mentored scanning in the Acute Medical Unit of a local hospital immediately after the course. Each trainer supervised four to six candidates. This, whilst effective for candidates, was difficult to organise. Trainers also found that six candidates were too many, citing the long time it took with each patient. Clearly, this was also unpleasant for the patients. Thus, this evolved into half-day supervised scanning sessions provided within the ICU of several hospitals. For these sessions, each trainer was able to supervise up to five candidates. We utilised a pragmatic approach to scanning, splitting the required skills into probe handling (including image optimisation) and image interpretation. Most sessions were attended by candidates with a mix of experience levels. First, the trainer assessed each candidate’s probe handling skills (if not already known). Upon satisfactory probe handling skills, each subsequent patient was only scanned by at most two candidates, and image interpretation was done by all candidates. Thus, all candidates got all the scans logged, even if they might not have physically manipulated the probe. The FAMUS pathway stipulates “recommended” number of scans, and both BTS and RCEM POCUS pathways do not require a fixed number of scans. As an approach, this also acknowledges prior experiences, and strikes a three-way balance between candidate needs, patient inconvenience, and trainer time. Using this model, each trainer was able to supervise up to ten lung scans per candidate, making a maximum of fifty supervised scans per half-day session. This means for most pathways, three half-day sessions are sufficient for a candidate to complete the requisite number of scans and triggered assessment. This model has been successfully implemented in several hospitals in the region.

For the indirectly supervised scans, initially, this was done as an in-person meeting between trainer and candidate. However, this was difficult to arrange. Some trainers did this asynchronously, reviewing scans on the US machine and then providing feedback to the candidate via text or email. This also took several communication points, which became difficult to keep track of. We then explored remote mentoring, and currently use SonoClipShare.com to facilitate asynchronous remote mentoring. This remote mentoring platform allows candidates to upload anonymised scans and send them to a named trainer for feedback and comments. Using this approach, a trainer need not be present for the scan, but will be able to provide ongoing mentoring and teaching to the candidate. Each full scan usually takes between five to ten minutes to review and provide comments on.

Responding to feedback from courses (Table 4), several changes were made. First, pre-course learning material was made available to reduce the load of lectures and prioritise practical scanning. Second, lectures were optimised to focus on scan protocols and pathologies, and physics was minimised and shifted to pre-course learning material.

### A collective commitment to learning

Unfortunately, there is no centralised database for POCUS trainers in the UK. The details of FUSIC trainers are stored in the Intensive Care Society database but it is not searchable. FAMUS supervisors are still searchable at the time of writing, from the Society of Acute Medicine website. RCEM, like BTS, have devolved their training to local hospitals, negating the need for a centralised database. Yet, as highlighted, there are many similarities across the accreditation pathways, and much overlap between the skills of POCUS practitioners across different specialties. Recognising a collective commitment to learning and training, we employed several strategies amongst candidates and trainers.

First, candidates are encouraged to contact our faculty to be put in touch with trainers at their local hospitals. In the UK, rotational training of doctors results in candidates having difficulties searching for trainers when they rotate to a new hospital every three to six months. We maintain a database of trainers across the region, which is used to connect candidates and trainers. Second, we opted to recognise the skills of trainers from the different accreditation pathways. Their competencies are further confirmed through the pairing of trainers on our courses described above. Thus, supervision can be facilitated by trainers from a different accreditation body, with oversight of a main trainer from the candidate’s chosen pathway. This method also allows candidates to be trained by a multidisciplinary selection of trainers within the region, exposing them to different styles of teaching, allowing them to pick up a variety of techniques, and increasing the likelihood of successful accreditation. Third, our database allows for triggered assessments in the chosen pathways, even if it requires travel to a different hospital. Maintaining such a network helps to mitigate the initial paucity of trainers in a particular pathway and encourages interdisciplinary relationships. We currently have over fifty active trainers across the accreditation pathways within the region, and there are ongoing plans to systematically collect a complete dataset for the Northwest of England and develop this into a searchable regional database. Such a network is vital for ensuring the resilience of the educational endeavour [18].

## Discussion

Lung POCUS is becoming an increasingly important aspect of intensive care medicine, acute and respiratory medicine, and emergency medicine. Yet, POCUS training continues to be of varying availability and quality. We report a multidisciplinary, multi-centre, and multi-pronged approach to lung POCUS accreditation. The courses have continued to receive very positive feedback, with participants appreciating the multidisciplinary aspects. In 2023, each trainer within our core faculty has supported the accreditation of at least five candidates each.

Several factors contributed to the success of this educational intervention. First, there is a small number of enthusiastic and dedicated trainers who have consistently given up their time, expertise, and experience to work together for POCUS training in the region. Second, the strong network of POCUS trainers and practitioners within the region and across specialties helped to mitigate the initial bottleneck of lack of mentors. Equally, the multidisciplinary approach to POCUS for the courses and supervision helped to build upon existing relationships between specialties. Third, the multi-pronged approach was responsive to feedback and observations within the region and enabled us to design courses and programmes that are fit for purpose. Fourth, there is increasing demand for POCUS training amongst trainees, and a large cohort of trainees across several specialties who are interested in pursuing accreditation. Fifth, the multidisciplinary approach to our POCUS training leverages different skill sets and allows efficient use of educational resources. It also captures a diverse range of candidates and forges multidisciplinary links which we believe are increasingly important for our increasingly complex patient population.

There are strengths to this report. It considers lung POCUS as an entire pathway and provides support using different methods depending on the stage of the pathway candidates are at. The multi-pronged approach to accreditation utilises an established educational process. It helped us to focus on examining and improving each step of the entire pathway (Table 1). Our educational approach builds links across multiple specialties, which aligns with wider multidisciplinary aims in healthcare [19]. There are plans to include physiotherapists into the trainer network since there have been high accreditation rates in the region.

Several limitations persist. There may be bias in the respondents of the initial survey since the respondents were mostly from an Intensive Care Medicine background. However, other teams have corroborated our findings in different countries and specialties [20, 21]. We have not followed every single candidate up through their accreditation processes. Since candidates are rotational and are spread across the entire region and beyond, it is difficult to obtain this data. In addition, we do not have access to interrogate such data for FUSIC or FAMUS, and pathways such as BTS and RCEM POCUS only require local sign-off, so it is challenging to obtain individual information. We plan to collect more robust data as a region in the future. Unfortunately, our database of trainers is not complete, but plans are in place to systematically identify trainers in the region. There is no validated way to maintain POCUS skills, and no agreed method for continuing professional development. Therefore, measurement of knowledge and skill retention continues to be difficult and stands out as a research priority.

There are several wider concerns about lung POCUS training. The presence of several different accreditation pathways makes it difficult for candidates to choose which is most appropriate for themselves and their professional development. Most trainers in our region do not have dedicated Supporting Professional Activities time to deliver training. There are discrepancies between the support given at trust, society, faculty, royal college, and national levels. For example, RCEM has advocated for Supporting Professional Activities time to support POCUS training across the country. Trainers therefore should get allowances within their job plans to deliver POCUS training. This is not the case for the Society of Acute Medicine or Intensive Care Society for FAMUS and FUSIC respectively. As a result, the burden of POCUS training is high considering the multiple steps of each accreditation pathway. As POCUS modalities become more diverse, the pathways may struggle to keep pace with innovation, and in turn, the burden caused by multiple accreditation modules will increase for candidates. There is therefore a need to explore single accreditation pathways across specialties, and maintain a robust, up-to-date, and searchable database which conforms to the General Data Protection Regulation principles.

These notwithstanding, POCUS training will continue to gain importance in medical practice, and innovative educational methods as described here should be shared to enable a more streamlined process with higher rates of successful accreditation. There are several plans which will help to gather more robust data on POCUS training in the region.

## Conclusion

We have described how adopting a multidisciplinary, multi-centre, and multi-pronged approach, utilising an established educational process (e.g. Action Learning), can help to improve lung ultrasound accreditation rates across a variety of pathways. Focusing on relational and collaborative processes are vital for the success of complex ultrasound educational endeavours. Further work needs to be done on systematically collecting data for more robust evidence.

### Supplementary Information

Supplementary Material 1.

## Data Availability

All data generated or analysed during this study are included in this published article.

## Abbreviations

AIM: Acute Internal Medicine
BTS: British Thoracic Society
COVID19: Sars CoV-2 or Coronavirus 2019
EM: Emergency Medicine
FAMUS: Focused Acute Medicine Ultrasoud
FICE: Focused Intensive Care Echocardiography
FUSIC: Focused Ultrasound in Intensive Care
ICM: Intensive Care Medicine
ICU: Intensive Care Unit
POCUS: Point-of-care ultrasound
RCEM: Royal College of Emergency Medicine
SPA: Supporting Professional Activities
US: Ultrasound
